# Improving the emission efficiency of MBE-grown GaN/AlN QDs by strain control

**DOI:** 10.1186/1556-276X-6-611

**Published:** 2011-12-02

**Authors:** Lang Niu, Zhibiao Hao, Jiannan Hu, Yibin Hu, Lai Wang, Yi Luo

**Affiliations:** 1 Department of Electronic Engineering, Tsinghua National Laboratory for Information Science and Technology, Tsinghua University, Beijing 100084, People's Republic of China

**Keywords:** GaN QDs, quantum-confined stark effect, internal quantum efficiency

## Abstract

The quantum-confined stark effect induced by polarization has significant effects on the optical properties of nitride heterostructures. In order to improve the emission efficiency of GaN/AlN quantum dots [QDs], a novel epitaxial structure is proposed: a partially relaxed GaN layer followed by an AlN spacer layer is inserted before the growth of GaN QDs. GaN/AlN QD samples with the proposed structure are grown by molecular beam epitaxy. The results show that by choosing a proper AlN spacer thickness to control the strain in GaN QDs, the internal quantum efficiencies have been improved from 30.7% to 66.5% and from 5.8% to 13.5% for QDs emitting violet and green lights, respectively.

## Introduction

Recently, with progress in the growth of high-quality bulk AlN [1,2], a lot of efforts have been devoted to GaN/AlN quantum dots [QDs] because of their unique properties such as broad emission wavelength range covering the whole visible light, which provides a promising way to achieve white light-emitting diodes [LEDs] [3]. Besides, the large conduction band offset (approximately 2 eV for GaN/AlN) offers a prospect to cover the fiber optical telecommunication wavelength range (1.3 to 1.55 μm) by intersubband transition [4,5].

By controlling the growth conditions, the sizes and densities of the GaN/AlN QDs can be varied, and the photoluminescence [PL] wavelength can also be tuned. However, the large lattice mismatch between GaN and AlN and their polarization properties induce a strong built-in electric field, causing a remarkable quantum-confined stark effect [QCSE] which reduces the internal quantum efficiency [IQE] of the QDs. The reason is that the built-in electric field leads to energy band decline and separation of electron and hole wave functions, resulting in the decrease of recombination efficiency as well as the red shift of emission wavelength. Furthermore, the emission peak shifts to a shorter wavelength with increasing injection current, which is caused by Coulomb screening of the internal electric field [6]. This phenomenon also exists in InGaN/GaN materials. In order to suppress the influence of QCSE, the compressive strain in the QD structures should be decreased, whereas, on the other hand, a certain degree of strain is required to perform the Stranski-Krastanov [S-K] mode growth of QDs. Therefore, it is a crucial issue to control the strain distribution in order to improve the IQE of GaN/AlN QD emission.

Nowadays, some work has been done to avoid the QCSE. Adelmann et al. grew self- assembled cubic GaN QDs by using plasma-assisted molecular-beam epitaxy [PA-MBE] on cubic AlN [7]. However, due to the very narrow growth window, it was difficult to grow high-quality GaN bulk and QDs. Cros et al. reported GaN/AlN QD growth on a-plane 6H-SiC [8,9]. This method suffered from an extremely expensive substrate, and compared with the GaN and AlN bulks grown on c-plane, the crystal quality still needed to be improved. Furthermore, an AlGaN buffer layer has been used instead of AlN to reduce the polarization effect [10]. However, a certain surfactant was required in order to achieve the two-dimensional to three-dimensional [2D-to-3D] growth transition [11]. Also, the bandgap of AlGaN is smaller than that of AlN; thus, the strong confinement in GaN QDs is weakened.

In our previous experiments, GaN/AlN QDs with varied morphologies have been obtained by properly choosing the growth parameters. The emission peaks of the QDs vary from 400 to 670 nm, and the QDs with a larger average height exhibit a longer emission wavelength but with a lower efficiency, due to the influence of QCSE. In this work, the morphologies and emission properties of GaN QDs grown on a partially relaxed AlN layer are investigated. The emission efficiencies of GaN QDs have been obviously improved by controlling the strain status of the underneath AlN layer.

## Experiments

**Figure 1:**
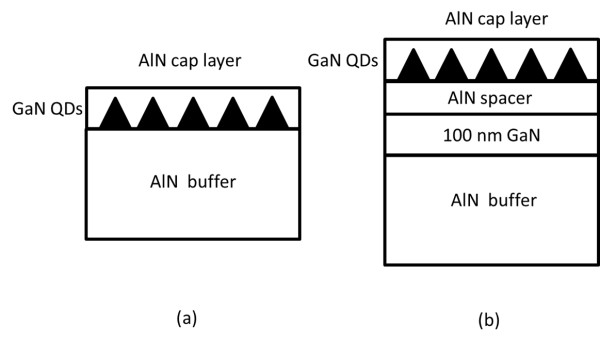
**Schematics of the (a) conventional and (b) proposed epitaxial structures of GaN/AlN QDs**.

In order to improve the emission efficiency of GaN/AlN QDs, we propose a novel epitaxial structure to control the strain in GaN QDs by inserting a GaN layer under the AlN barrier layer. The epitaxial structures of GaN/AlN QDs are illustrated in Figure 1. For the proposed QD structure, as shown in Figure 1b, a 100-nm-thick GaN insertion layer is grown above the AlN buffer, followed by an AlN spacer with varied thickness, then GaN QDs, and an AlN cap layer. The GaN insertion layer is designed to be partially relaxed; hence, the strain status of the following AlN spacer and GaN QDs can be controlled by varying the thickness of the AlN spacer.

The samples were grown on c-plane sapphire substrates by PA-MBE. Reflection high-energy electron diffraction [RHEED] and optical reflection spectrum were used to monitor the growth *in situ*. The Al and Ga sources were supplied by conventional Knudsen effusion cells. Two sets of samples were prepared with different AlN spacer thicknesses. Control samples, with a conventional structure as shown in Figure 1a, were grown without the GaN insertion layer. Table 1 summarizes the structural parameters of the samples. One set of samples emits violet light (around 400 nm), while the other set of samples grown under higher substrate temperature emits green light (around 520 nm). Samples without the AlN cap layer were also prepared for morphology measurement.

**Table 1:** Structural parameters of the GaN/AlN QD samples and the measured morphology characteristics

Sample	AlN spacer thickness (nm)	GaN insertion layer thickness (nm)	QD density (cm^-2^)	Mean QD height (nm)	Mean QD diameter (nm)	QD AR
A	N/A	None	4.4 × 10^11^	1.1	10.2	0.11
B	60	100	4.0 × 10^11^	1.5	10.5	0.14
C	40	100	2.2 × 10^11^	1.9	11.8	0.16
D	20	100	9.6 × 10^10^	2.9	17.2	0.17

The samples' surface morphologies were measured by scanning electron microscopy [SEM] and atomic force microscopy [AFM]. The crystalline properties were examined by transmission electron microscopy [TEM] and X-ray diffraction [XRD]. To evaluate the samples' optical properties, temperature-dependent PL measurements were carried out using a 325-nm laser as excitation.

## Results and discussion

**Figure 2:**
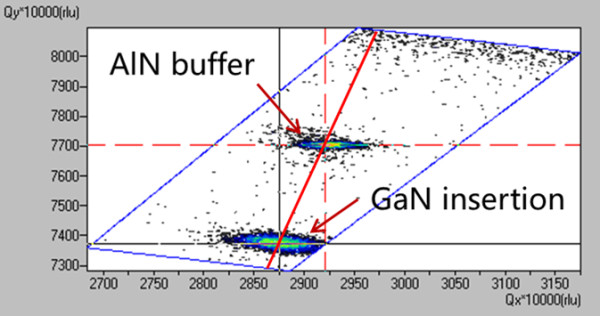
**XRD reciprocal space mapping measurement result of a typical sample**.

**Figure 3:**
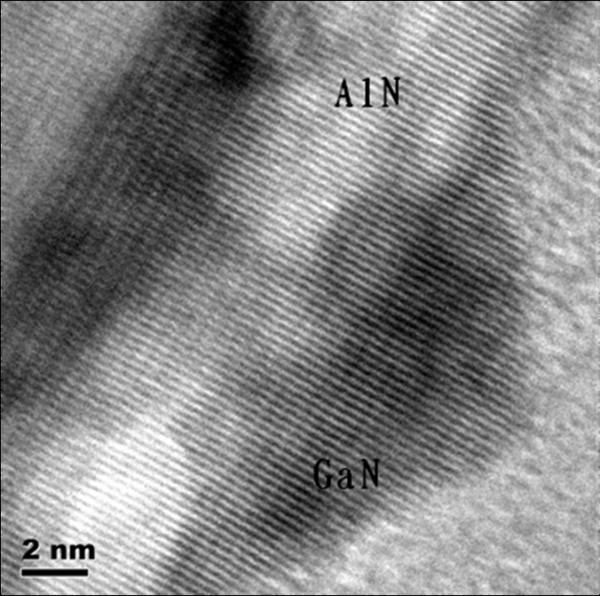
**High-resolution cross-sectional TEM image of the GaN/AlN QDs**.

As mentioned above, a partially relaxed GaN insertion layer is introduced in order to control the strain status of the GaN QDs. Figure 2 shows the typical XRD reciprocal space mapping measurement result of the samples. The relaxation factor can be defined as (*a*_GaN-epi_-*a*_AlN-bulk_)/(*a*_GaN-bulk _-*a*_AlN-bulk_), where *a*_GaN-epi _is the in-plain lattice constant of the GaN insertion layer, and *a*_GaN-bulk _and *a*_AlN-bulk _are the standard in-plain lattice constants of GaN and AlN bulks [12]. According to the XRD reciprocal mapping data, the relaxation factor of the GaN insertion layer is calculated to be 63.3%, which manifests that the insertion layer is partially relaxed, and the in-plain lattice constant of the top GaN is larger than that of the AlN bulk. Then, the subsequently grown AlN spacer is under tensive strain, and the strain status varies according to its thickness, i.e., the thicker AlN spacer is with a smaller in-plain lattice due to relaxation. The high resolution cross-sectional TEM image of the GaN/AlN QDs is shown in Figure 3, which reveals that the GaN QDs are grown coherently with the lattice of the AlN layer and have the same in-plain lattice constant with the AlN spacer. Therefore, by varying the thickness of the AlN spacer, the strain of GaN QDs can be controlled; thicker AlN spacer results in a larger strain in GaN QDs.

**Figure 4:**
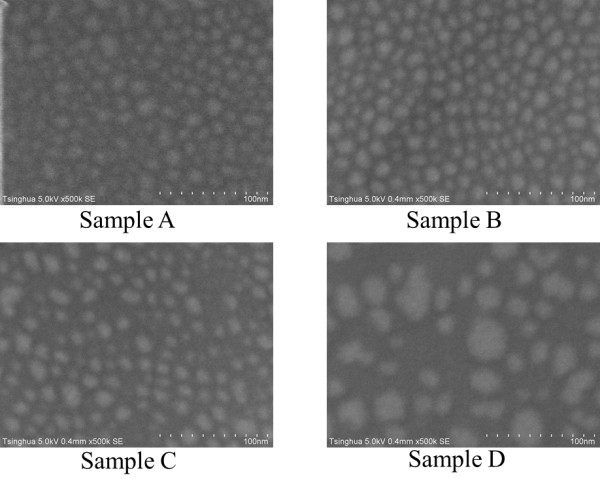
**SEM images of the GaN QD samples**.

Initially, the samples emitting violet light are analyzed. Among the four samples described in Table 1, according to the aforementioned structural properties, the GaN QDs in sample A have the largest strain, while the QDs in sample D have the smallest strain. As observed from the RHEED patterns for S-K growth of GaN QDs, it takes 18, 25, 30, and 35 s for samples A, B, C, and D, respectively, to complete the 2D-to-3D transition. This indicates that from samples A through D, the critical thickness for QD formation increases orderly due to the reduction in strain, and as a result, larger and higher dots will be formed because of more GaN accumulated during the process. As shown in Figure 4, the SEM measurement reveals that the average QD diameter increases obviously from samples A through D, along with the decreased QD density. According to the AFM measurements, the mean QD heights of samples A, B, C, and D are 1.1, 1.5, 1.9, and 2.9 nm, respectively. These morphology characteristics are summarized in Table 1.

**Figure 5:**
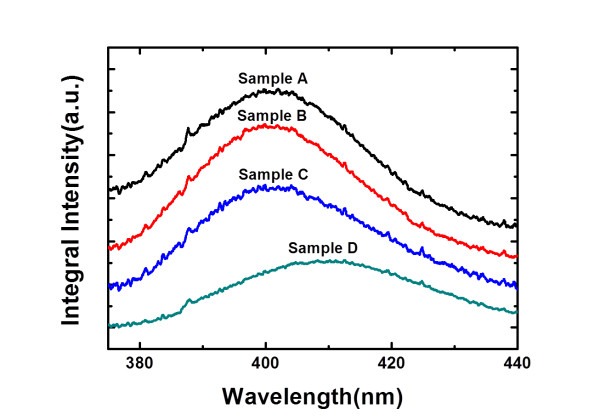
**Room-temperature PL spectra of the GaN QD samples**.

**Figure 6:**
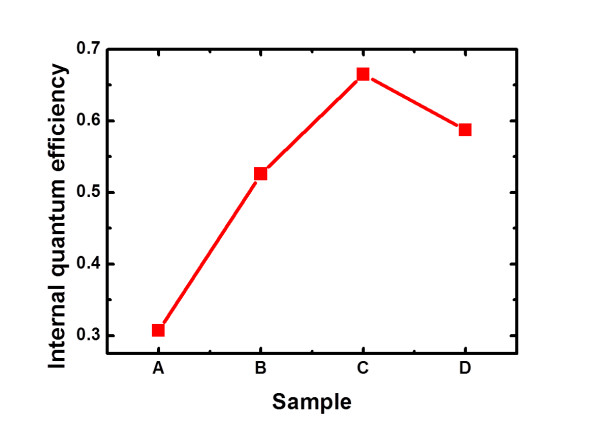
**The IQEs of the samples obtained by temperature-dependent PL measurements**.

Figure 5 shows the room-temperature PL spectra of the four samples. The emission peaks of samples A, B, and C are all approximately 400 nm, while the PL peak of sample D exhibits a small red shift. By performing the temperature-dependent PL measurement from 4 K to 300 K, the IQE of the QDs can be calculated by the ratio of the integral PL intensity at 300 K to that at 4 K, and the results are shown in Figure 6. It can be seen that the IQE of sample A with conventional structure is 30.7%, and the IQE increases to 66.5% for sample C with a 40-nm thickness of AlN spacer. As for sample D which has the thinnest AlN spacer of 20 nm, the IQE drops a little to 58.7%.

There are two factors when considering the influence of QCSE on the IQE of the QDs: one is the strain-induced internal electric field, and the other is the QD morphology. A careful simulation is required to fully understand the influence of QD morphology. Ngo et al. reported that the emission wavelength of QDs exhibits a red shift with the increasing QD height, base, volume, or aspect ratio [AR] at a fixed volume [13]. For our samples, as seen in Table 1, the QD diameter, aspect ratio, and volume increase with the QD height. Therefore, in order to simplify the discussion, we only consider the QD height in the following part.

**Figure 7:**
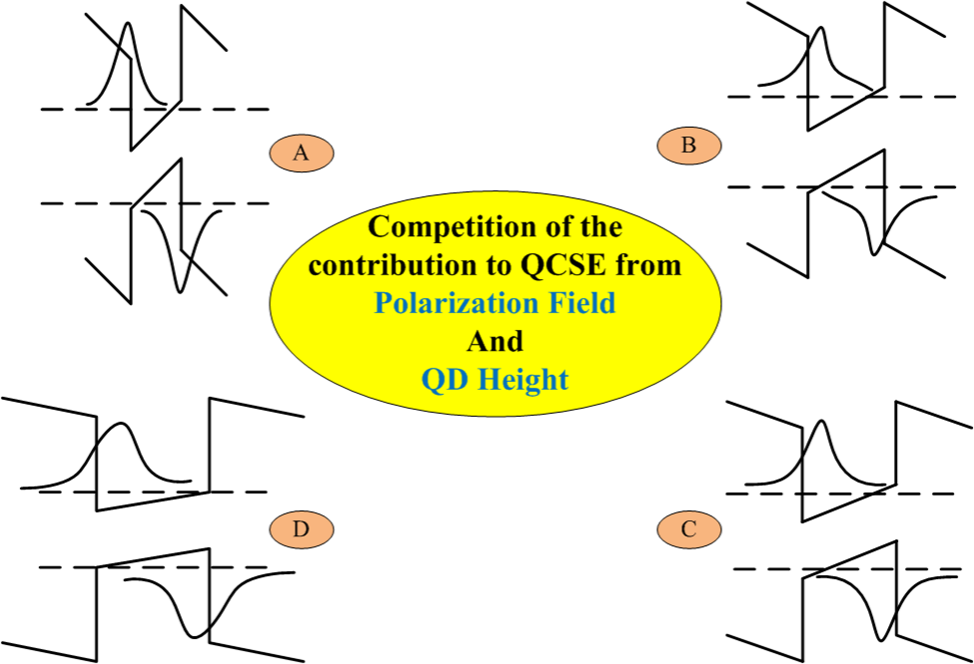
**Illustration of the effects of internal electric field and QD height on the GaN QDs**.

For GaN/AlN QDs, when either the internal electric field or the QD height increases, the IQE will decrease due to the reduction of the electron-hole overlap [14]; at the same time, a red shift of the emission peak can be observed. On the contrary, reduction in either of these two factors will lead to improvement of the emission efficiency and blueshift of the emission peak. For samples from A through D, the internal electric field decreases, while the QD height increases; the ultimate effects depend on which factor is dominant. The fact that the emission efficiency increases from samples A through C means that for these samples, the effect on emission efficiency from the reduction in internal electric field overcomes that from the QD height. On the other hand, samples A, B, and C exhibit almost the same emission peak wavelength. This is because the increase of QD height and the decrease of internal electric field balance out the influence on the emission wavelength. As for sample D, the emission efficiency drops and the emission peak red shifts about 10 nm, due to the large QD height playing a dominant role. How these two factors affect the IQE is illustrated in Figure 7.

This mechanism also accounts for the improvement of the samples emitting green light. The IQE of the sample without the GaN insertion layer is only 5.8%, while for the sample with the proposed epitaxial structure, the IQE has been improved to 13.5%. These results imply a promising way to optimize the performance of QD LEDs.

## Conclusions

GaN/AlN QD samples with a partially relaxed GaN insertion layer followed by an AlN spacer layer have been grown by PA-MBE. The proposed structure can control the strain in GaN QDs and thus the QCSE induced by polarization. As a result, the IQEs for GaN QDs emitting violet and green lights have been improved from 30.7% to 66.5% and from 5.8% to 13.5%, respectively. And for the samples with the AlN spacer of a certain range of thickness, the emission wavelength keeps nearly unchanged when the IQE increases.

## Competing interests

The authors declare that they have no competing interests.

## Authors' contributions

LN wrote, conceived, and designed the experiments. LN, ZBH, JNH, and YBH grew the samples and analyzed the data. LN, LW, and YL did all the measurements. All authors discussed the results, contributed to the manuscript text, commented on the manuscript, and approved its final version.
